# Polymorphisms of glutathione-S-transferase M1, T1, P1 and the risk of prostate cancer: a case-control study

**DOI:** 10.1186/1756-9966-28-32

**Published:** 2009-03-05

**Authors:** Monika Sivoňová, Iveta Waczulíková, Dušan Dobrota, Tatiana Matáková, Jozef Hatok, Peter Račay, Ján Kliment

**Affiliations:** 1 Department of Medical Biochemistry, Comenius University, Jessenius School of Medicine, Malá hora 4, 036 01, Martin, Slovakia; 2 Department of Nuclear Physics and Biophysics, Division of Biomedical Physics, Comenius University, Faculty of Mathematics, Physics, and Informatics, Mlynská dolina F1, 842 48, Bratislava, Slovakia; 3 Department of Urology, Comenius University, Jessenius School of Medicine and University Hospital, Kollárova 2, 036 59, Martin, Slovakia

## Abstract

**Background:**

It has been suggested that polymorphisms in glutathione-*S*-transferases (GST) could predispose to prostate cancer through a heritable deficiency in detoxification pathways for environmental carcinogens. Yet, studies linking *GST *polymorphism and prostate cancer have so far failed to unambiguously establish this relation in patients. A retrospective study on healthy, unrelated subjects was conducted in order to estimate the population *GST *genotype frequencies in the Slovak population of men and compare our results with already published data (GSEC project-Genetic Susceptibility to Environmental Carcinogens). A further aim of the study was to evaluate polymorphisms in *GST *also in patients with prostate cancer in order to compare the evaluated proportions with those found in the control subjects.

**Methods:**

We determined the *GST *genotypes in 228 healthy, unrelated subjects who attended regular prostate cancer screening between May 2005 and June 2007 and in 129 histologically verified prostate cancer patients. Analysis for the *GST *gene polymorphisms was performed by PCR and PCR-RFLP.

**Results:**

We found that the *GST *frequencies are not significantly different from those estimated in a European multicentre study or from the results published by another group in Slovakia. Our results suggest that *Val/Val *genotype of *GSTP1 *gene could modulate the risk of prostate cancer, even if this association did not reach statistical significance. We did not observe significantly different crude rates of the *GSTM1 *and *GSTT1 *null genotypes in the men diagnosed with prostate cancer and those in the control group.

**Conclusion:**

Understanding the contribution of *GST *gene polymorphisms and their interactions with other relevant factors may improve screening diagnostic assays for prostate cancer. We therefore discuss issues of study feasibility, study design, and statistical power, which should be taken into account in planning further trials.

## Background

Prostate cancer is the most common cancer among men in industrialized countries with the main risk factor being the age of over 50. Prostate cancer is uncommon in men younger than 45, but becomes more common with increasing age. The average age at the time of diagnosis is 65 [1-4]. Since early detection increases the chance of successful treatment, the prostate-specific antigen (PSA) test and the digital rectal examination should be offered to men annually beginning at age 50. Men with high risk should begin testing at age 45. The only well-established risk factors for prostate cancer are age, ethnicity, geography and family history of prostate cancer. However, research in the past few years has shown that genetic, socioeconomic and environmental factors, particularly diet and lifestyle, likely have an effect as well. It is assumed that increased exposure to procarcinogens and carcinogens contained in tobacco smoke, debris, fermented food, polluted water, air etc., is implicated in multistage carcinogenesis. Therefore, the assessment of the hazard of prostate cancer coming from the pollution of the environment is of increasing importance. Moreover, the differences in the effectiveness of detoxification/activation of carcinogens may help us understand why one man may be at a higher risk than another [3].

Glutathione-S-transferase (GST) are phase II enzymes which are responsible for catalyzing the biotransformation of a variety of electrophilic compounds, and have therefore a central role in the detoxification of activated metabolites of procarcinogens produced by phase I reactions [5]. The GSTM1, GSTT1 and GSTP1 members of the multigene family are candidate cancer-predisposing genes. The relation of polymorphisms in these genes to chemical carcinogenesis has been extensively studied in various populations. Several population-based studies have reported prevalence ranging from 47% to 58% for the *GSTM1 *deletion genotype and from 13% to 25% for the *GSTT1*-null genotype among white Europeans [1,6]. For *GSTP1*, the prevalence rates of *Ile/Val *heterozygosity and *Val/Val *homozygosity were found to be between 38% to 45.7% and 7% to 13% respectively [7].

GST deficiencies may increase the risk of somatic mutation, which subsequently leads to tumor formation [6]. The absence of GSTM1 activity is caused by the inheritance of two null alleles (alleles that have a deletion of the *GSTM1 *gene). Similarly, individuals with no GSTT1 activity also have inherited null alleles of the *GSTT1 *gene. A single nucleotide polymorphism in the *GSTP1 *gene causes the substitution of isoleucine for valine at amino acid codon 105 (Ile^105^Val), which substantially diminishes GSTP1 enzyme activity and lessens the effective capacity for detoxification [8,9]. However, the published data about the association of *GST *polymorphism and susceptibility to prostate cancer are controversial. Some studies suggest that the *GSTM1, GSTT1 *and *GSTP1 *polymorphisms are associated with prostate cancer susceptibility [10,11], whereas other studies report no association [12,13].

The aim of this study was twofold: 1) to estimate the prevalence of the *GSTM1, GSTT1 *and *GSTP1 *gene polymorphisms in the Slovak population of men and compare those results with the respective data published by other groups (GSEC project – Genetic Susceptibility to Environmental Carcinogens); and 2) to evaluate the frequencies of the *GSTT1 *and *GSTM1 *null genotypes and polymorphisms in *GSTP1 *also in the patients with prostate cancer in order to compare the evaluated proportions with those found in the controls.

## Methods

### Case description

The present study was performed under the approval of the Ethical Boards of Jessenius School of Medicine, Comenius University and the informed written consent was obtained from all individuals prior to their inclusion in the study.

Blood samples from 228 subjects (median age of 63, IQR 56–70 years) were obtained from healthy, unrelated subjects living in the north-western part of Slovakia, who were invited to attend the Department of Urology for regular prostate cancer screening between May 2005 and June 2007. The second part of the study was designed as a case-control study (approximately two controls per one case). The criteria for selecting patients were based on a clinical proforma, covering medical, pathological and histopathological records. A total of 129 prostate cancer patients (median age of 70, IQR 63–74 years) who were histologically verified as having prostate cancer were invited to participate in the project. Patients who had a first-degree relative (brother or father) with a confirmed diagnosis of prostate cancer were excluded in order to avoid familial prostate cancer cases. The samples were used for estimating *GST *gene frequencies.

Both patients and controls were interviewed regarding age, smoking habits, possible chemical exposure, previous and/or current prostate diseases, and incidence of cancer and chronic diseases. The individuals were grouped in never-smokers and ever-smokers. The studied population is described in Table 1.

**Table 1:** General characteristic of the control and prostate cancer patient groups

	**Control group** Number (%) of subjects	**Prostate cancer patients** Number (%) of subjects
No.	228	129

Smoking status		

Smokers	51 (22%)	35 (27%)

Non-smokers	177 (78%)	94 (73%)

PSA (ng/ml, means ± SD)	2,73 ± 6,78	30,46 ± 77,89***

*** p < 0.001

### Chemicals

Proteinase K was obtained from AppliChem (DE). All the primers, chemicals used for PCR and restriction enzyme, were purchased from Eppendorf (USA). All other chemicals used for DNA isolation were purchased from Sigma Co. (USA).

### Genotyping

Peripheral venous blood was collected in 10 ml heparinized tubes and the specimens were immediately stored at -20°C for genotyping. From both, cases and controls, genomic DNA was isolated from peripheral leukocytes by proteinase K digestion, phenol/chloroform extraction and ethanol precipitation, dissolved in TE buffer (pH 7.5) and stored at -20°C until genotype analysis.

**Figure 1:**
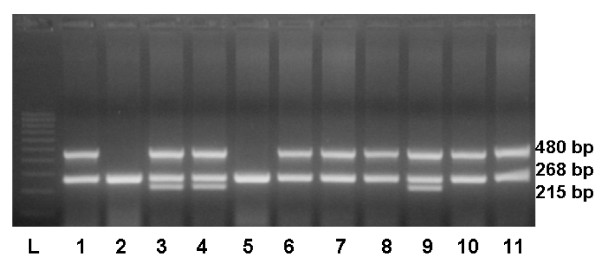
**Detection of polymerase chain reaction (PCR) amplification of *GSTT1 *(480 bp fragment), *β-globin *(268-bp fragment) and *GSTM1 *(215-bp fragment) genes**. Absence of the PCR product indicates the null genotype. Ethidium bromide-stained electrophoresed representative PCR products samples: 100 bp ladder (lane L); absence of null genotypes (lanes 3, 4, 9); *GSTT1*-null allele (lanes 2, 5) and *GSTM1*-null allele (lanes 1, 2, 5, 6, 7, 8, 10, 11).

A multiplex polymerase chain reaction (PCR) method was used to detect either the presence or absence of *GSTM1 *and *GSTT1 *genes in the genomic DNA samples simultaneously in the same tube; *β-globin *gene was co-amplified and used as an internal control [14]. This technique does not distinguish between heterozygote and homozygote *GSTM1*- and *GSTT1*-positive genotypes, but it does conclusively identify the null genotype [15]. Genomic DNA (100 ng) was amplified in a total volume of 25 μl reaction mixture containing 25 pmol of each *GST *primers (GSTM1: forward 5'-GAA CTC CCT GAA AAG CTA AAG C-3' and reverse 5'-GTT GGG CTC AAA TAT ACG GTG G-3', GenBank accession no. NM_146421; GSTT1: forward 5'-TTC CTT ACT GGT CCT CAC ATC TC-3' and reverse 5'-TCA CCG GAT CAT GGC CAG CA-3', GenBank accession no. NM_000853); 25 pmol β-globin gene primers (forward 5'-CAA CTT CAT CCA CGT TCA CC-3' and reverse 5'-GAA GAG CCA AGG ACA GGT AC-3'); 200 μmol/l deoxynucleoside triphosphates; 1 U of Taq polymerase in 10 × PCR buffer composed of 16.6 mmol/l (NH_4_)_2_SO_4 _and 20.0 mmol/l MgCl_2_, pH 8.8. After initial denaturation for 3 min at 94°C, 39 cycles were performed for 1 min at 94°C (denaturation), for 1 min at 60°C (annealing) and for 1 min at 72°C (extension), followed by a final step for 5 min at 72°C. The *GSTM1 *(215-bp), *GSTT1 *(480-bp) and *β-globin *(268-bp) amplified products were visualized by electrophoresis on ethidium-bromide-stained 3% agarose gel (Fig. 1). For deletions of *GSTM1 *and *GST1 *no amplified products can be observed, whereas the *β-globin *specific fragment confirms the presence of amplifiable DNA in the reaction mixture.

**Figure 2:**
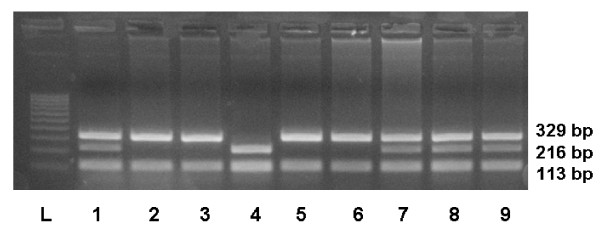
**Cleavage of 442 bp PCR products of *GSTP1 *gene by the *Alw26I *restriction endonuclease**. Ethidium bromide-stained electrophoresed representative PCR-RFLP products samples: 100 bp ladder (lane L), *Ile/Ile *allele (lanes 2, 3, 5, 6); *Ile/Val *allele (lanes 1, 7, 8, 9) and *Val/Val *allele (lane 4).

The *GSTP1 Ile*^105^*Val *substitution was detected using the PCR-RFLP approach as the substitution by guanine introduced restriction site that can be recognized by an endonuclease *Alw26I*. PCR reactions were performed in a total volume of 25 μl of solution containing 10 × PCR buffer (16.6 mmol/l (NH_4_)_2_SO_4_, 20.0 mmol/l MgCl_2_, pH 8.8, 1.2 μl DMSO, 1.2 μl DTT), 200 μmol/l deoxynucleoside triphosphates, 1 U of Taq DNA polymerase, 100 ng of genomic DNA and 25 pmol of *GSTP1 *primers (forward 5'-GTA GTT TGC CCA AGG TCA AG-3' and reverse 5'-AGC CAC CTG AGG GGT AAG-3', GenBank accession no. NM_000852). The reaction started for 3 min at 94°C, followed by 5 cycles of PCR (cycle 1: 94°C for 15 s, 64°C for 30 s, and 72°C for 1 min) during which the annealing temperature decreased by 1°C for each cycle. This step was followed by 30 cycles of denaturation (for 15 s at 94°C), annealing (for 30 s at 59°C), and extension (for 1 min at 72°C). A final polymerization step (for 5 min at 72°C) was carried out to complete the elongation process and yield a 442-bp fragment. A negative control (PCR without template) was included in each set of PCR reactions. Each PCR product (10 μl) was digested for 4 hours with the restriction enzyme Alw26I (5 U) and electrophoresed on ethidium-bromide-stained 1.5% agarose gel. The presence of the *Ile/Ile *allele was detected by 329-, and 113-bp fragments, whereas the *Val/Val *allele was confirmed by 216-, and 113-bp fragments. The heterozygote *Ile/Val *allele was characterized by fragments consisting of 329, 216, and 113 bp (Fig. 2) [7].

### Statistical analysis

Age is presented as median and interquartile range (IQR) because the data showed departures from normality (according to Shapiro-Wilk's test). The χ^2 ^method was used to test frequencies of genotypes/allele in prostate cancer patients and controls. The strength of the nominal association in the contingency tables is reflected by Cramér's (V) coefficient of contingency. The odds ratios (OR), estimates of the relative risk, with 95% confidence intervals (CI) were computed to assess strengths of association of the genotypes with prostate cancer. All p values cited are two-sided alternatives; differences resulting in a p value of less or equal to 0.05 were declared statistically significant [16]. The Hardy Weinberg equilibrium was tested for the genotype proportions in the control group, as a measure for quality control.

## Results

Since previous reports suggested that there are no differences in *GSTM1, GSTT1 *and *GSTP1 *allele frequencies in relation to age and sex [17], we conducted a retrospective study on a selected population of men in order to examine whether the gene frequencies were consistent with research findings across Europe. Statistical analysis of data collected from a survey of community sample in the north-western part of Slovakia showed that our estimates were not significantly different from either those found in the Caucasian population of Garte and co-workers [1] (Table 2) or those found previously by a research group in Slovakia [1] (Table 3).

**Table 2:** Distribution of *GSTP1*, *GSTT1 *and *GSTM1 *genotypes in our control group and in Caucasian population (GSEC project-Genetic Susceptibility to Environmental Carcinogens) published by Garte and co-workers [1].

**Polymorphism**	**Our control group** Number (%) of subjects	**Caucasians-GSEC** Number (%) of subjects	**95% CI for proportion difference**	**Cramér's V**	**p-value**
***GSTP1***					

No.	228	1137			

Ile/Ile	110 (48.2)	498 (43.8)	-0.03 to 0.12	0.033	0.22

Ile/Val+Val/Val	118 (51.8)	561 (49.3)	-0.05 to 0.09	0.018	0.51

***GSTT1***					

No.	228	5577			

positive	183 (80.3)	4774 (80.2)			

null	45 (19.7)	1103 (19.8)	-0.05 to 0.06	0.005	0.99

***GSTM1***					

No.	228	10514			

positive	98 (43.0)	4931 (46.9)			

null	130 (57.0)	5583 (53.1)	-0.03 to 0.10	0.011	0.24

**Table 3:** Distribution of *GSTT1 *and *GSTM1 *genotypes in our control group and in Slovak population (GSEC project-Genetic Susceptibility to Environmental Carcinogens) published by Garte and co-workers [1].

**Polymorphism**	**Our control group** Number (%) of subjects	**Slovak population-GSEC** Number (%) of subjects	**95% CI for proportion difference**	**Cramér's V**	**p-value**
***GSTT1***					

No.	228	332			

positive	183 (80.3)	272 (82.0)			

null	45 (19.7)	60 (18.0)	-0.05 to 0.09	0.021	0.62

***GSTM1***					

No.	228	332			

positive	98 (43.0)	162 (48.8)			

null	130 (57.0)	170 (51.2)	-0.03 to 0.14	-0.057	0.18

Among our control group, genotype frequencies did not deviate significantly from the Hardy-Weinberg equilibrium [p = 0.72 (GSTP1), p = 0.8 (GSTT1) and p = 0.43 (GSTM1)].

Because the published data about the association of *GST *polymorphism and susceptibility to prostate cancer are not conclusive, and because it was suggested that the incidence of prostate cancer varies with geography, the second purpose of the study was to analyze the strength of these associations in our selected population. Calculated chi-square for equality of mean column scores and Cramér's V yielded 0.506 and 0.023, respectively, which did not account for significant differences in the *GST *frequencies between healthy subjects and those diagnosed with prostate cancer. The absence of any association between null genotypes or polymorphism in *GST *and prostate cancer was confirmed also by analyzing case-control groups. Table 4 shows the distribution of the *GST *genotypes among controls and prostate cancer patients. The patients did not have significantly different frequencies in genotypes and alleles in comparison to controls.

**Table 4:** Distribution of *GSTP1*, *GSTT1 *and *GSTM1 *genotypes in controls and patients with prostate cancer.

**Polymorphism**	**Controls** Number (%) of subjects	**Cases** Number (%) of subjects	**95% CI for proportion difference**	**Cramér's V**	**OR (95% CI)**	**p-value**
***GSTP1***						
				
No.	228	129				

Ile/Ile	110 (48.2)	56 (43.4)			1.0	

Ile/Val+Val/Val	118 (51.8)	73 (56.6)	-0.15 to 0,06	0.047	0.72 (0.45 to 1.13)	0.38

Val/Val	5 (2.2)	6 (4.7)	-0,08 to 0,01	0.068	2.17 (0.54 to 9.18)	0.22

						

***GSTT1***						
				
No.	228	129				

positive	183 (80.3)	105 (81.4)			1.0	

null	45 (19.7)	24 (18.6)	-0.08 to 0.09	-0.014	0.93 (0.51 to 1.66)	0.80

						

***GSTM1***						
				
No.	228	129				

positive	98 (43.0)	60 (46.5)			1.0	

null	130 (57.0)	69 (53.5)	-0,07 to 0,14	0.034	0.87 (0.55 to 1.37)	0.52

In addition, we have found no clear association between smoking habits and prostate cancer, and between smoking habits and single or combined genotypes in relation to prostate cancer. Neither did the comprehensive score, a pooled value indicating the presence of at least one variant allele, show a significantly reduced or unchanged risk of prostate cancer (data not shown).

## Discussion and evaluation

To assess possible association between *GST *gene polymorphisms and occurrence of prostate cancer in Slovakia, we had to infer from population estimates acquired in the first part of the study on a sample of 228 consecutive men who scheduled appointments in the Department of Urology.

It is known that the allele frequencies of metabolic genes are not equally distributed throughout the human population but follow diverse ethnic and/or geographic-specific patterns. Our results on *GSTM1*- and *GSTT1*-null frequencies, 57% and 19.7%, respectively, did not differ significantly either from the values obtained previously by a Slovakian group of researchers (51.2% and 18%, respectively) or from those published by other authors [1]. The prevalence rate of *Ile/Val *heterozygosity and *Val/Val *homozygosity was 51.8% in our control subjects. This frequency is also similar to the frequencies found in other studies that analyzed *GSTP1 *polymorphism [18-20].

Some studies have reported a relationship between *GST *variants and risk of prostate cancer [9,10,12,13,21]. Investigation of the *GSTP1 *gene did not reveal any significant association between heterozygous *GSTP1 *genotype (*Ile/Val*) and prostate cancer. However, our results suggest that *Val/Val *genotype of *GSTP1 *gene could modulate the risk of prostate cancer, even if this association did not reach statistical significance. It should be kept in mind that the inability to reject the null hypothesis could be due to low power of the test because of a relatively small sample size. Therefore, the lack of significance does not necessarily mean equality of the distributions. It is plausible that polymorphism at the *GSTP1 *locus can play an important role in the susceptibility to different types of cancer. Association of the *GSTP1 Val *allele with cancer could be expected since the conversion of the amino acid at codon 105 from isoleucine to valine substantially lowers activity of the altered enzyme. It has been predicted from molecular modelling that the amino acid at this site lies in a hydrophobic binding site for electrophile substrates and thus affects the substrate binding [22]. On the other hand, there are also studies which did not prove any independent effect of this type of polymorphism on the susceptibility for prostate cancer [23-25].

In the present study, we did not observe significantly different crude rates of the *GSTM1 *and *GSTT1 *null genotypes in the men diagnosed with prostate cancer and those in the control group. Our data and the data published by other research groups suggest that differences in the *GST *frequencies between prostate cancer patients and the control group are relatively small, which therefore makes it difficult to separate the groups from each other based on statistical data analysis. Once again, the high variability in the groups could mask statistical differences due to low power. The easiest way to improve precision is to increase the number of subjects and patients in the experimental design. However, this may not be applicable to all research conditions due to such factors as additional costs, poorer availability of resources, lower population, which compromises the number of subjects eligible for investigation. In order to achieve a power of at least 80%, we have to identify other explanatory variables and the control for them, and/or apply meta-analysis in order to increase sample size.

Recent studies on *GST *polymorphism have also evaluated the combined effect of *GSTM1 *and *GSTT1 *genotypes, but most of them failed to show any significant association between the joint deficiency of these genes and prostate cancer risk [24,26]; to our knowledge, only one study has reported a significant increase prevalence of prostate cancer among carriers of both *GSTM1 *and *GSTT1 *null genotypes [27]. Some studies, which combined data from other genotypes, have shown that the concurrent lack of *GSTM1/GSTT1 *and *GSTP1 *genes posed a significantly increased risk of prostate cancer [20,28,29]. However, these studies have not been confirmed by other authors [23]. One of the reasons for such discrepancy in the findings might lie in the difficulty of analyzing the impact of the modified GST activity on detoxification of known carcinogens. GST has overlapping substrate specificities; therefore, deficiency of a single GST isoenzyme may be compensated by other isoforms. Another important factor is the differential expression of genes for GST in different cells.

The variation in published prostate cancer prevalence rates can be attributed partly to methodological differences in survey design, including age distribution of the population surveyed. It is also known that the incidence of prostate cancer is underestimated, maybe due to poor compliance of elderly with screening recommendations. Thus, regular follow-ups are difficult to achieve and, as a consequence, many men never know they have prostate cancer. It has been reported that the calculated prevalence of prostate cancer at death (i.e. histological evidence) for a 60-year-old man is 32%, whereas but the prevalence in living men (clinically-defined disease) is approximately 4% [30].

In contrast to the possible role of GST in environmental carcinogenesis, it has been suggested that *GST *genotypes conferring lower enzyme activity may be of advantage for the patients who are undergoing chemotherapeutic treatment for neoplastic disease because reduced detoxification potentially enhances effectiveness of cytotoxic drugs [31]. Although somewhat speculative, the *GST *polymorphisms might be a protective factor during the period of chemotherapy, as the carriers of *GST *null genotypes might better respond to the treatment. At present, it is difficult to confidently evaluate the *GST *polymorphisms impact on prostate cancer patients. Apparently, it would be far too simplistic to attribute a complex problem such as prostate cancer to any single cause. Although it is methodologically difficult to identify and separate all the factors that make it difficult to identify individual changes, it is nevertheless possible to conduct a carefully designed international and/or multicentric study, or of combining results of several independent studies on the topic.

## Conclusion

Our results suggest a possible association between the *GSTP1 Val/Val *genotype and the occurrence of prostate cancer. However, broad confidence intervals indicate a naturally high variability in *GST *polymorphisms in the population, which has given less weight to the observed differences in *GSTP1 Val/Val *genotype frequencies between the patients and the control subjects. Even if it was shown that our study was not designed and powered to detect single gene effects, as well as gene-environment interactions, we cannot exclude that inter-individual differences in GST enzyme activity mediated by polymorphic genes, and reflected in insufficient detoxification of environmental mutagens and carcinogens, may be involved in the pathway, ultimately leading to tumor formation. Because understanding of the contribution of *GST *gene polymorphisms and their interactions with other relevant factors may improve screening diagnostic assays for prostate cancer, as well as clinical management of the patients, further studies are needed to validate observed associations and to identify the causal sequence for prostate cancer from *GST *gene polymorphisms, providing it exists.

## Competing interests

The authors declare that they have no competing interests.

## Authors' contributions

MS, TM, JH, PR carried out *GST *polymorphism analysis and analyzed the data. MS, IW and DD wrote the manuscript, JK collected the samples and patient's clinical data. All authors read and approved the final manuscript.
